# Consequential Impacts of Tobacco Use on Cognitive Performance

**DOI:** 10.1093/geroni/igab046.3580

**Published:** 2021-12-17

**Authors:** Shandell Pahlen, Michael Stallings, Robin Corley, Sally Wadsworth, Chandra Reynolds

**Affiliations:** 1 University of California, University of California, California, United States; 2 University of Colorado, Boulder, Boulder, Colorado, United States; 3 University of Colorado Boulder, Boulder, Colorado, United States; 4 University of California Riverside, Riverside, California, United States

## Abstract

Tobacco use represents a pernicious lifestyle factor that may influence processes of aging, including cognitive functioning. As individuals tend to start smoking before adulthood, it may serve as an important factor in cognitive development and maintenance. We explored smoking history-cognition associations in a sample approaching midlife. Study data was derived from the Colorado Adoption/Twin Study of Lifespan behavioral development and cognitive aging (CATSLife 1; N = 1195 [53% F]; x̄age = 33.2 years, SD = 5.0). All cognitive measures were t-scored covering working memory, spatial reasoning, processing speed (WAIS-III Digit Span, Block Design, and Digit Symbol, and Colorado Perceptual Speed) and episodic memory domains (Picture Memory, immediate and delayed). Tobacco use measures included ever-smokers, current-smokers, and log-transformed packyears. Mixed-effects regression models were applied, accounting for sex, age, race, ethnicity, and clustering among siblings. Tobacco use was associated with worse episodic memory, spatial and speed performance, but not working memory. When educational attainment was included, patterns remained consistent though attenuated. Results suggested current-smokers scored 0.27 to 0.36 SD lower than non-smokers on speed and spatial reasoning tasks. Episodic memory performance was reduced by approximately 0.07 to 0.1 SD per log packyear. In a sample approaching midlife, the harmful impacts of tobacco use on cognitive performance may be already apparent with cumulative impacts of packyears on episodic memory and current smoking associated with spatial and speed performance. This work helps to elucidate the temporal associations of an important lifestyle factor that may influence cognitive functioning prior to midlife.

